# Supplementary figures and images for: Cell-type- and chromosome-specific chromatin landscapes and DNA replication programs of Drosophila testis tumor stem cell–like cells
Source: Genome Res. 2026 Jan;36(1):83–101. doi: 10.1101/gr.280809.125 (PMC12758400; doi:10.1101/gr.280809.125)

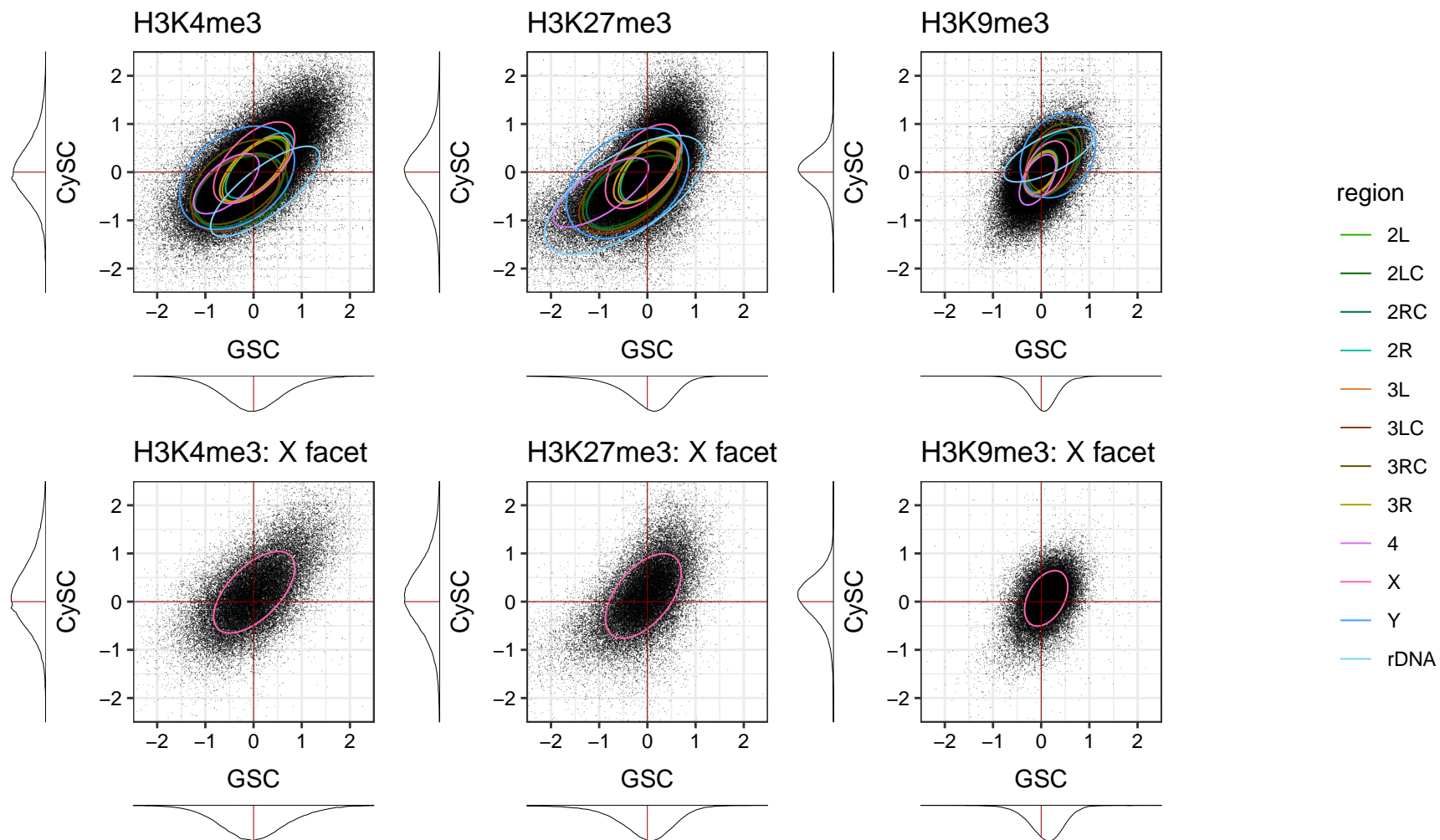

Supplement: Supplement 8 [file Supplemental_Code.zip › Upd-Germline-Genomics-main/illustrations/ChromatinCellTypeScatter.pdf]

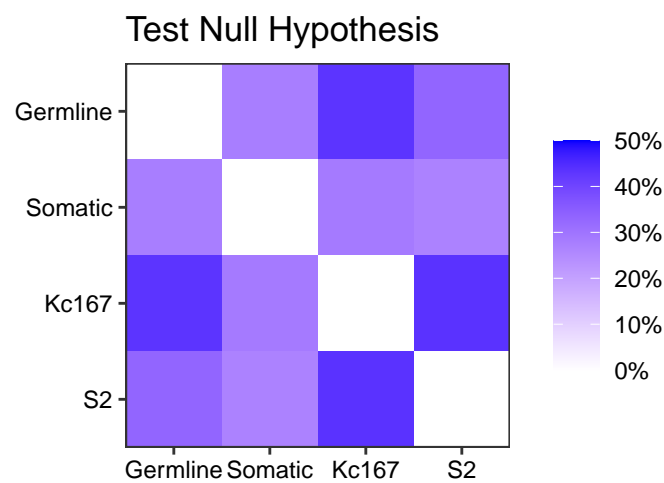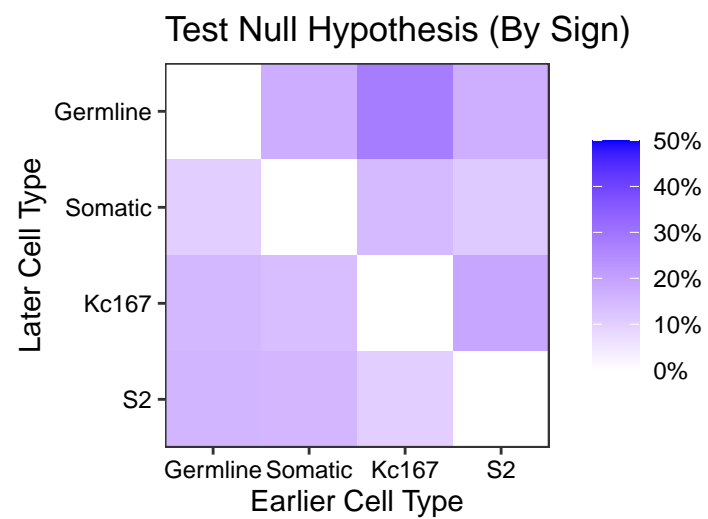

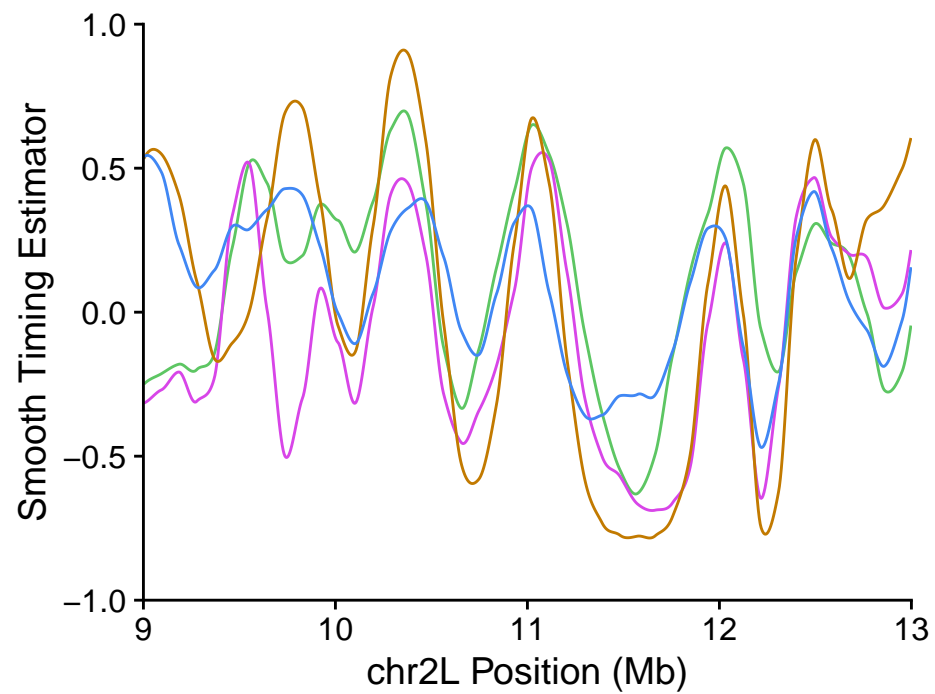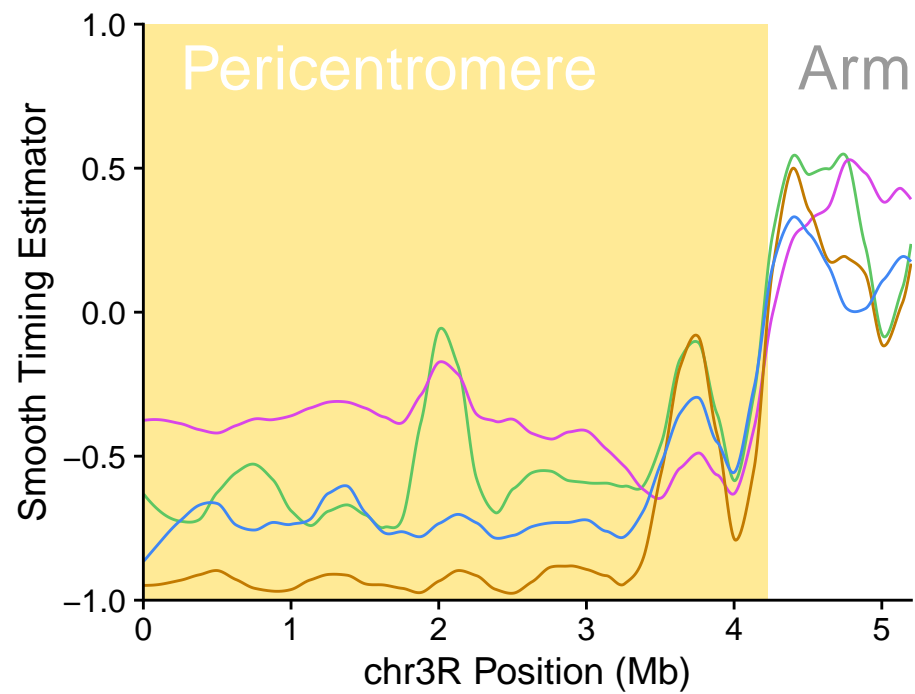

## Cluster Dendrogram

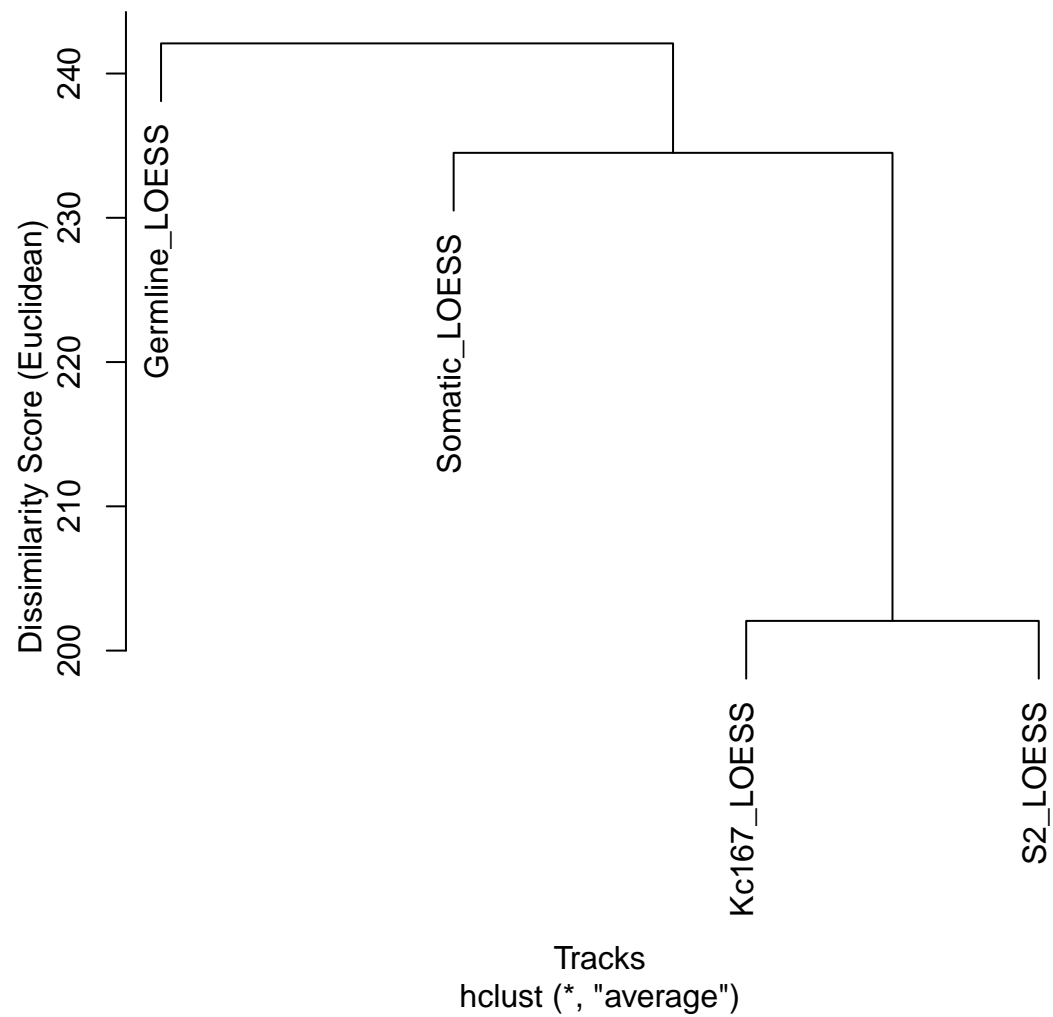

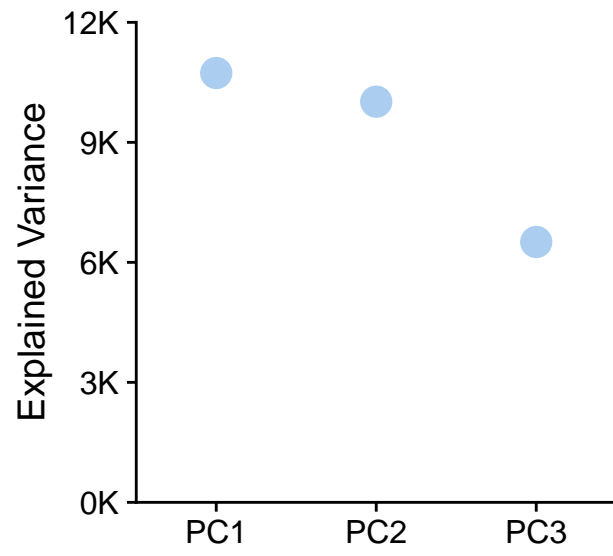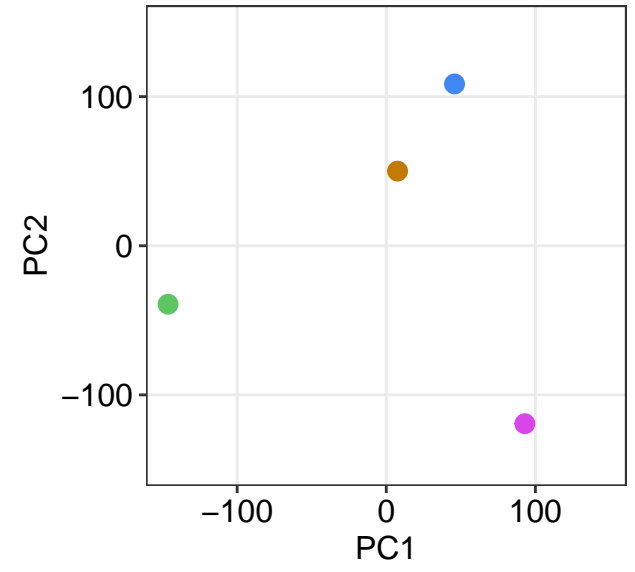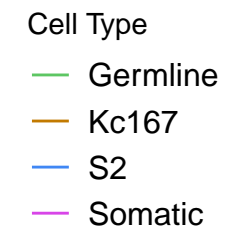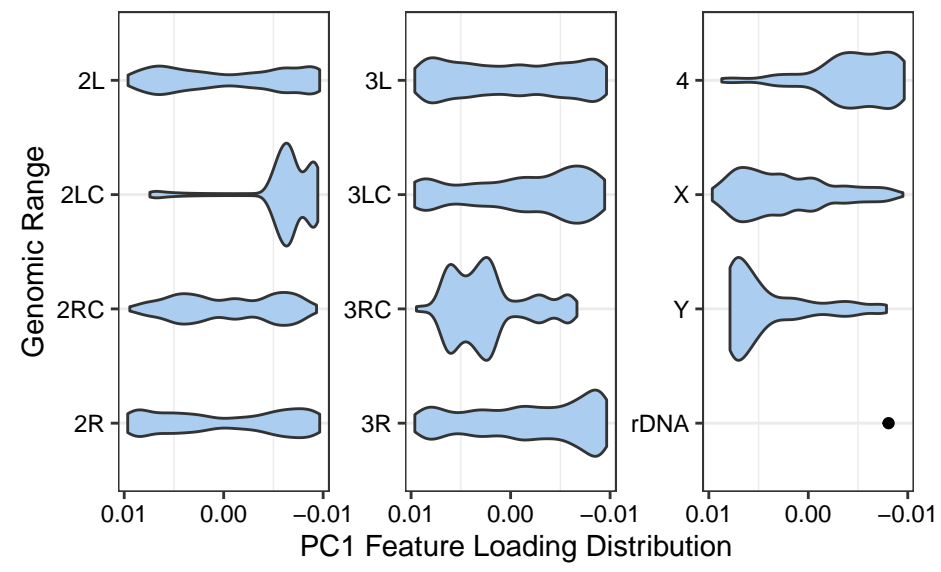

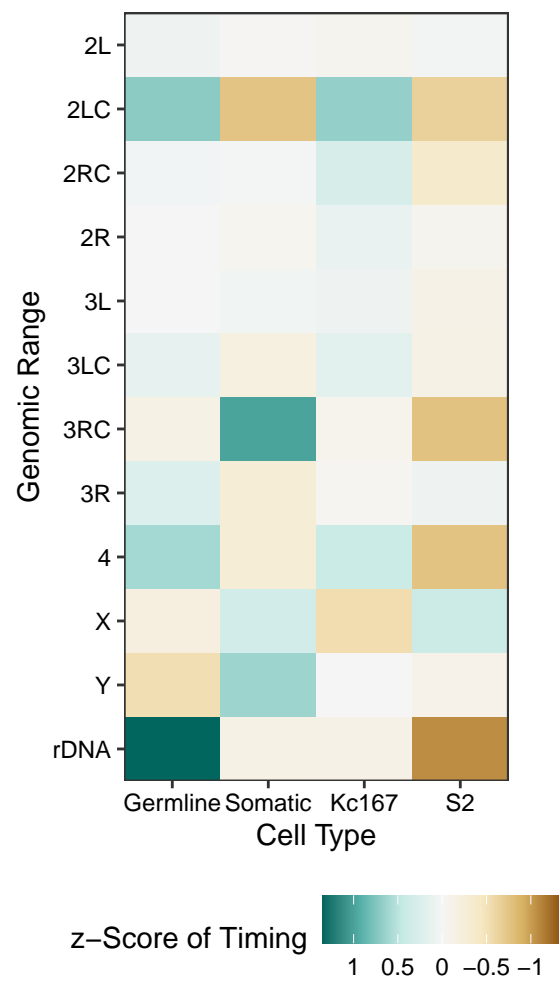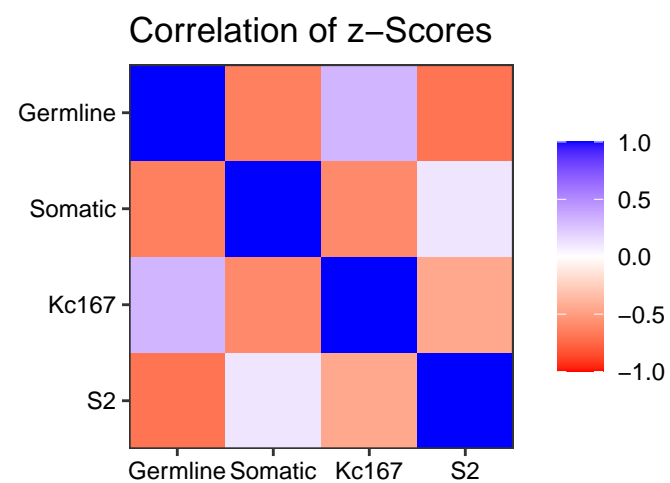

Supplement: Supplement 8 [file Supplemental_Code.zip › Upd-Germline-Genomics-main/illustrations/Fig6Panels.pdf]
